# Contrast Sensitivity Testing in Retinal Vein Occlusion Using a Novel Stimulus

**DOI:** 10.1167/tvst.9.11.29

**Published:** 2020-10-27

**Authors:** Shubhendu Mishra, Nenita Maganti, Natalie Squires, Prithvi Bomdica, Divya Nigam, Arthur Shapiro, Manjot K. Gill, Alice T. Lyon, Rukhsana G. Mirza

**Affiliations:** 1 Department of Ophthalmology, Northwestern University, Chicago, IL, USA; 2 American University, Washington, DC, USA

**Keywords:** contrast sensitivity, retinal ischemia, visual adaptation

## Abstract

**Purpose:**

This study evaluated a novel tool known as the motion diamond stimulus (MDS), which utilizes contrast-generated illusory motion in dynamic test regions to determine contrast sensitivity (CS).

**Methods:**

Patients with treated unilateral retinal vein occlusions (RVOs) underwent three assessments: the MDS, the Pelli-Robson (PR), and the National Eye Institute's Visual Function Questionnaire (VFQ-25). The MDS assessment produced two data end points, α and β. The α value represents the overall contrast threshold level and the β value serves to quantify the adaptability of the visual contrast system. The CS parameters from the MDS and log CS PR output values were used to compare RVO eyes (*n* = 20) to control eyes (*n* = 20).

**Results:**

The study participants had a mean composite VFQ-25 score of 89.5 ± 10.4 on the VFQ-25. A significant difference was observed between the RVO eyes and the control eyes in PR log CS scores (*P* value = 0.0001) and in MDS α value (*P* value = 0.01). No difference in MDS β value was found between the study groups (*P* value = 0.39).

**Conclusions:**

The results for the MDS assessment's α parameter corroborated the PR scores, suggesting contrast sensitivity threshold impairment in patients with RVO. No significant difference in β value was observed, suggesting that adaptability of the visual system is maintained in treated RVO eyes.

**Translational Relevance:**

Currently, visual complaints cannot be entirely identified by Snellen visual acuity alone. The MDS offers potentially a more complete look at visual function, by including contrast sensitivity and may be able to quantify changes otherwise overlooked in retinal disease progression.

## Introduction and Background

Retinal vein occlusion (RVO) is the second most common cause of vision loss due to vascular retinal disease.^1^ The progression of RVO is typically monitored through a combination of visual acuity (VA) measurements using the Snellen chart and optical coherence tomography (OCT).^2^ In treating patients with RVO, ophthalmologists are often faced with patients reporting vision changes that cannot be quantified by VA measurements or imaging. Therefore, visual-function tests, such as contrast sensitivity (CS) assessment, can provide valuable information to evaluate the progression of RVO and the impact of treatment. CS is a fundamental aspect of vision. Its measurement can provide useful information about a patient's visual function beyond VA. CS is a strong predictor of real-world performance (e.g. driving performance, mobility and walking speed, postural stability, and falls).^3^ CS measurements have demonstrated value in the detection and progression of many other retinal diseases, including diabetic retinopathy and age-related macular degeneration.^4^^,^^5^ Although there are limited baseline data on the CS of patients with RVO as compared to controls, CS measurements have demonstrated value in assessing ischemic retinal diseases and have characterized visual changes in RVO post-treatment.^6^^,^^7^ In order to better characterize vision loss in RVO, a more robust understanding of CS changes in RVO is necessary.

Several CS tests with good psychometric properties have been developed that are easily administered.^8^ The most widely used test is the Pelli-Robson (PR) CS Chart.^9^ Although the PR is the gold standard for CS research, it is rarely used clinically due to its physical size, practicality, and scientific disagreement regarding rules of use.^10^

Due to the limitations of the PR CS assessment, an additional CS assessment currently in the research domain known as the motion diamond stimulus (MDS) was tested in this study.^11^ The MDS, developed at American University, is a physically stationary diamond that appears to move perpetually in one of four directions (up, down, right, and left). “Movie 1” in Flynn and Shapiro's 2018 open-source publication demonstrates this perpetual motion.^12^ The MDS assessment consists of three components: a center diamond, four edges of the diamond, and a surround field. The center diamond has a fixed mid-level luminance; the surround is a circular field with a luminance of two hertz; and the four edges are thin rectangular bars surrounding the diamond that also have a luminance modulating at two hertz. The direction of the perceived motion of the diamond is dependent on the temporal phase of the edge's modulations relative to the phase of the surround modulation.^12^

The principle behind MDS is that motion energy can be created by changing the luminance contrast among shape, the edge, and the background.^13^^–^^17^ The key to creating perpetual motion in one direction is the luminance level of the edges. If the luminance of the edges is fixed at black and white, then the motion of the shape will shift back and forth.^18^ However, if the luminance of the edges also changes in time, the motion can be made to shift perpetually in one direction.^12^^,^^19^^,^^20^ The direction of motion depends on the phase of the edges’ modulations relative to the luminance changes of the shape or surrounding field. The physiological processes underlying the perception are established because the contrast changes produce first-order motion energy in the stimulus.^21^^,^^22^ Healthy visual systems can perceive this motion with remarkable precision (less than 1 second of visual angle in optimal conditions).^12^

The MDS is a useful digital tool for assessing vision because it translates changes in luminance contrast into the perception of motion, and it does so without any physical displacement of the lights. It allows observers to make a simple force-choice task, which is to pick the direction of the diamonds: up, down, right, or left. Furthermore, the MDS has multiple modifiable parameters (edge widths, contrast modulation ratio of the edges, and the surround) that can characterize a gamut of vision changes with a graphical output comparing the observer's contrast threshold (i.e. contrast modulation at which observers correctly identified the direction of motion 80% of the time) versus the contrast ratio between edge and field modulation.

The MDS assessment's primary output values are contrast thresholds (akin to PR's log CS score output) that identify finite thresholds at which an individual can perceive contrast. In the MDS, however, the contrast threshold is not a single value, but rather a function of the contrast ratio. Contrast ratio is defined as the ratio between the background contrast magnitude and the contrast level of the edges in the MDS assessment. In 2012, Flynn and Shapiro demonstrated that global motion is determined by local phase difference.^22^ For the visual system to continuously perceive motion during the MDS test, the visual system must adapt to local changes in contrast to ensure that contrast signals remain in range. Increasing the contrast ratio (by 2, 4, 8, and 16 times) creates increasing challenges for the visual adaptive system. Although a healthy eye should not be affected by contrast modulation ratio changes, impaired visual systems would find these changes difficult.^12^ Graphically, the adaptability of the visual contrast system can be represented by the slope of the contrast threshold (dependent variable) plotted against the contrast ratio (independent variable). A slope of zero indicates that contrast ratio changes had no effect on visual performance, whereas a non-zero slope indicates that contrast ratio changes altered visual performance in the MDS assessment.

Nigam et al. obtained normative data from 95 healthy students at American University substantiating the MDS assessment and showing that as contrast modulation ratios increase, the observers’ ability to correctly identify direction of motion decreases (Nigam D, et al. 2018, unpublished). There are ongoing clinical trials for applying the MDS assessment to patient populations with eye diseases, such as central serous retinopathy. It has been hypothesized that eye disease will adversely impact the observer's ability to identify the motion of the diamond stimulus and lead to characteristic findings in the MDS output.

Squires et al. also obtained additional normative data for the MDS assessment at Northwestern Memorial Hospital from patients with no ocular diseases that analyzed the impact of pupillary dilation on CS (Squires N, et al. 2020 ARVO E-abstract 3366503). This study demonstrated that pupillary dilation led to alternations in CS that could be detected by both the PR and the MDS assessments (Squires N, et al. 2020 ARVO E-abstract 3366503).

In this study, we will measure the CS capabilities of patients treated with anti-vascular endothelial growth factor (VEGF) for RVO (both central retinal vein occlusion [CRVO] and branched retinal vein occlusion [BRVO]) utilizing both the MDS and PR assessments. We will compare the results of both assessments to control eyes. We hypothesize that the MDS will serve as a reliable outcome measure to understand the changes in visual function in these patients beyond what is captured by VA and the PR.

## Methods

### Study Design

This study was a cross-sectional assessment of 20 patients with unilateral RVO (either branch or central RVO) receiving treatment for retinal edema at Northwestern Memorial Hospital. The protocol was approved by the institutional review board at Northwestern University. All patients provided informed consent before participation in the study.

### Eligibility

Patient recruitment took place in the Northwestern ophthalmology clinics between December 2018 and July 2019. Patients above the age of 18 years, with best corrected visual acuity (BCVA) 20/40 or better in both eyes and less than or equal to 2+ nuclear sclerosis or cortical cataracts, who had a prior central or branch RVO in one eye (study eye) but were unaffected in the other eye (non-study) were considered for this study. Patients were receiving ongoing treatment with anti-VEGF intravitreal injections. Exclusion criteria included BCVA worse than 20/40 in either eye, asymmetric cataracts, or other retinal conditions, including diabetic retinopathy, age-related macular degeneration, macular hole, retinal detachment or tear, and epiretinal membrane. Patients that met these criteria were invited and subsequently consented to participate in the study by a team member.

### Study Visit

Study participants underwent three assessments during their study visit: the PR, the MDS, and the National Eye Institute's Visual Functioning Questionnaire-25 (NEI VFQ-25). Patients were undilated before beginning these assessments and patients were given the option to take the assessment with or without their corrective lenses depending on their preference.

The PR CS chart was tested on a patient's right eye followed by the left eye in a well-lit room. The chart was placed at 1 meter from the patient and the patients used a standard eye occluder to test one eye at a time. The patient was asked to read the lowest line they could see with their right eye and then asked to read the lowest line they could see backward with their left eye. The log CS score for each eye was gathered as one of the study end points.

Following the PR, the patients took the MDS assessment. Prior to starting the MDS assessment, all patients had been dark adapted for at least 30 minutes. A portable photometer was used to measure the ambient luminance in the testing room with the laptop on and all the room lights turned off. The laptop used to administer the MDS test was kept at 30% brightness to minimize any ambient light. Furthermore, the luminance of the monitor used as the MDS display screen, kept at 100% brightness, was also measured. The ambient luminance of the testing environment ranged from 2 to 4 Lux and the luminance of the MDS display screen ranged from 26 to 29 Lux for all study visits.

The entire MDS test was conducted with all the room lights turned off, except for the ambient light from the laptop used to control the program. The MDS display monitor was placed at 50 cm from the patient at their eye level. The MDS test was administered to the unaffected eye followed by the RVO affected eye for the first 10 patients. The order was reversed for the following 10 patients, with the RVO affected eye tested first followed by the unaffected eye. Because the MDS assessment has an initial learning curve, this reversal served to minimize bias that would falsely elevate the performance of the eye that was tested later. Each eye was tested individually, with the other eye being occluded. While taking the assessment, the patients were given no indication by the examiner on whether they were reading the directions of the diamonds correctly. The MDS assessment took approximately 15 minutes per eye. After completing the MDS test, the program output eight contrast threshold values. The first 4 values are the patient's contrast threshold at contrast modulation ratios of 2, 4, 8, and 16, all at edge-width 3. The last 4 values are also the patient's contrast threshold at ratios of 2, 4, 8, and 16, but at edge-width 6. These data served as the end points for the MDS assessment.

After completing the MDS assessment, patient-reported visual function was assessed with the NEI VFQ-25. The NEI VFQ-25 provided a composite score that quantified visual function.^15^ As a clarification, the VFQ-25 was not utilized to compare RVO eyes to control eyes, but rather to characterize the overall visual health of this study's participants.

After completing these assessments, no additional study-related follow-up was performed with the participants.

### Statistical Analysis

The statistical analysis was performed using Microsoft Excel and the statistical programming environment R (version 2.2.1). The study population characteristics in Table 1 are presented as means with 95% confidence intervals. Baseline differences between the study RVO eyes and control eyes were established using a paired *t*-test. Furthermore, a paired *t*-test was also utilized to examine the differences between study RVO eyes and control eyes using the PR log CS scores.

**Table 1.** The Study Population Characteristics

	Overall Cohort (*n* = 20)
**Age, y**	
Mean (SD)	62.9 y (15.64 y)
Median (IQR)	63.0 y (22.25 y)
**Gender, *n* (%)**	
Male	10 (50)
Female	10 (50)
**Race, *n* (%)**	
White	17 (85)
Black/African American	2 (10)
Hispanic	0
Other	1 (5)
**BMI**	
Mean (SD)	27.22 (5.22)
**Smoking status, *n* (%)**	
Former or current smoker	5 (25)
**Diabetes, *n*** (%)	2 (10)
**Hypertension, *n*** (%)	12 (60)
**Hyperlipidemia, *n*** (%)	13 (65)
**Months from RVO diagnosis to study visit**	
Mean (SD)	32 mo (29)
**Visual Function Questionnaire composite score**	
Mean (SD)	89.5 (10.4)

All data is presented as number of patients (n) out of the entire cohort (20), followed by the percent unless otherwise indicated.

IQR, interquartile range; BMI, body mass index.

MDS data end points for each patient were plotted as two separate graphs, one for each edge-width, with contrast modulation ratio on the x-axis and contrast threshold on the y-axis. Contrast threshold y-values were graphed on a log scale. Using Excel's GROWTH function, an exponential curve was calculated for each graph in the form y = αe^βx^, where α corresponds to the threshold level of the curve and β corresponds to the slope of the curve as a function of contrast ratio. After the α and β values were determined for each patient, the study RVO eye could be compared to the non-study eye using a one-tailed paired *t*-test for each set of variables α and β.

The NEI VFQ-25 scores were calculated according to published guidelines.^23^ The mean of all the NEI VFQ-25 subscales were used to calculate the overall composite score.

## Results

### Subject Characteristics

Twenty patients were included for a total of 20 treated RVO eyes (11 BRVO and 9 CRVO eyes) and 20 contralateral eyes as controls. Participants were equally distributed across sex (*n* = 10 women) and had a mean age of 62.9 years ± 15.64 (mean ± SD). The majority (85%) of participants were of Caucasian descent. The study population was additionally 10% African American and 5% Asian (categorized as “other”). Notably, 60% of the population had a diagnosis of hypertension and 65% with hyperlipidemia. On average, the study patients had been diagnosed with RVO 32 ± 29 months prior to participating in this study and were all receiving regular anti-VEGF injection treatment from their ophthalmologist. An average composite VFQ-25 score of 89.5 was calculated for the participants based on survey responses. Other normative characteristics are described in Table 1.

### Characteristics of the RVO and Control Eyes

Baseline ocular comparisons were performed using measurements from the most recent visit (see Table 2) for the treated RVO study eyes (*n* = 20) versus the control eyes (*n* = 20). The study eyes had a mean logarithm of the minimum angle of resolution (logMAR) BCVA of 0.129 ± 0.149, whereas control eyes all had a mean logMAR BCVA of 0.033 ± 0.067. There was a significant difference in VA between the study and control eyes (*P* value = 0.0123). No significant difference in IOP was found between study and control eyes (*P* value = 0.795). The majority of study and control eyes had either clear lenses or lenses with trace cataracts (60% for study eyes and 55% for control eyes). Thirty-five percent of study eyes and 40% of control eyes had 1 to 2+ cataracts, whereas 5% of both study and control eyes had 3+ cataracts. Of the study eyes, 55% were categorized as BRVO and 45% were classified as CRVO. On imaging, no significant difference in central foveal thickness (CFT) of the retina was found between the study and control eyes (*P* value = 0.2314 with mean study eye CFT = 286 µm versus mean control eye CFT = 273 µm).

**Table 2.** Baseline Ocular Comparison Between Study and Non-Study Eye

	Study Eye	Non-Study Eye
**Mean logMAR BCVA (SD)** ^a^	0.129 (0.149)	0.033 (0.067)
**IOP (mm Hg), mean (SD) Cataract, *n* (%)**	15.85 (2.91)	16.10 (3.13)
Clear lens	10 (50)	9 (45)
Trace NS	2 (10)	2 (10)
1 to 2+	7 (35)	8 (40)
3+	1 (5)	1 (5)
**Pseudophakia, *n* (%)**	3 (15)	2 (10)
**RVO Classification, *n* (%)**		
BRVO	11 (55)	
CRVO	9 (45)	
**Imaging data (at/before study visit)**		
CFT (µm), mean (SD)	286 (38)	273 (29)
Presence of intraretinal fluid	8 (40%)	0
Presence of subretinal fluid	1 (5%)	0

a Indicates that a *P* value comparing the study versus non-study eye for the marked parameter is significant and < 0.05.

All data is presented as number of eyes (*n*) out of the cohort (20), followed by a percent, unless otherwise indicated.

BCVA, best corrected visual acuity; IOP, intraocular pressure; BRVO, branched retinal vein occlusion; CRVO, central retinal vein occlusion; CFT, central foveal thickness.

### Comparison of RVO Study Eyes Versus Controls

As shown in Table 3, CS thresholds were evaluated for 20 treated RVO eyes and 20 fellow control eyes using the MDS assessment. With a three MOA edge-width, treated RVO eyes did not demonstrate a statistically significant difference in either overall threshold levels (α for treated RVO = 0.0276 vs. α for controls = 0.0141, *P* value = 0.1047) or slope of the thresholds as a function of contrast modulation ratios (β for treated RVO = 0.1127 vs. β for controls = 0.1109, *P* value = 0.4580). At a six MOA edge-width, treated RVO eyes did not demonstrate a statistically significant difference in the slope of the thresholds as a function of contrast modulation ratios (β for treated RVO = 0.1227 vs. β for controls = 0.1270, *P* value = 0.3901), but they did demonstrate a statistically significant increase in overall threshold level (α for treated RVO = 0.0010 vs. α for controls = 0.0061, *P* value = 0.0193).

**Table 3.** Mean Contrast Sensitivity With the MDS Test and Pelli-Robson Test by Group

Test	RVO (*n* = 20)	Control (*n* = 20)	*P* Value
MDS edge-width 3			
α (mean, SD)	0.0276 (.0534)	0.0141 (.0234)	0.1047
β (mean, SD)	0.1127 (.0748)	0.1109 (.0550)	0.4580
MDS edge-width 6			
α (mean, SD)	0.001 (0.0131)	0.0061 (.001)	0.0193*
β (mean, SD)	0.1227 (0.0748)	0.127 (0.0667)	0.3901
Pelli-Robson, log CS			
Mean (SD)	1.53 (0.1727)	1.695 (0.1385)	0.0001^*^

Quantitative data from the MDS and Pelli-Robson test. The symbol α represents the threshold level for the threshold versus contrast ratio curve and β represents the slope of the threshold versus contrast ratio curve.

* Indicates that the *P* value is significant and < 0.05.

The Figure graphically depicts the MDS data obtained from the study population by plotting mean threshold values (y-axis) versus contrast modulation ratios (x-axis) at two different edge widths (3 vs. 6 MOA). A best-fit exponential regression is shown for four groups of data (study eyes at edge-width 3, control eyes at edge width 3, study eyes at edge-width 6, and control eyes at edge with 6).

**Figure.**
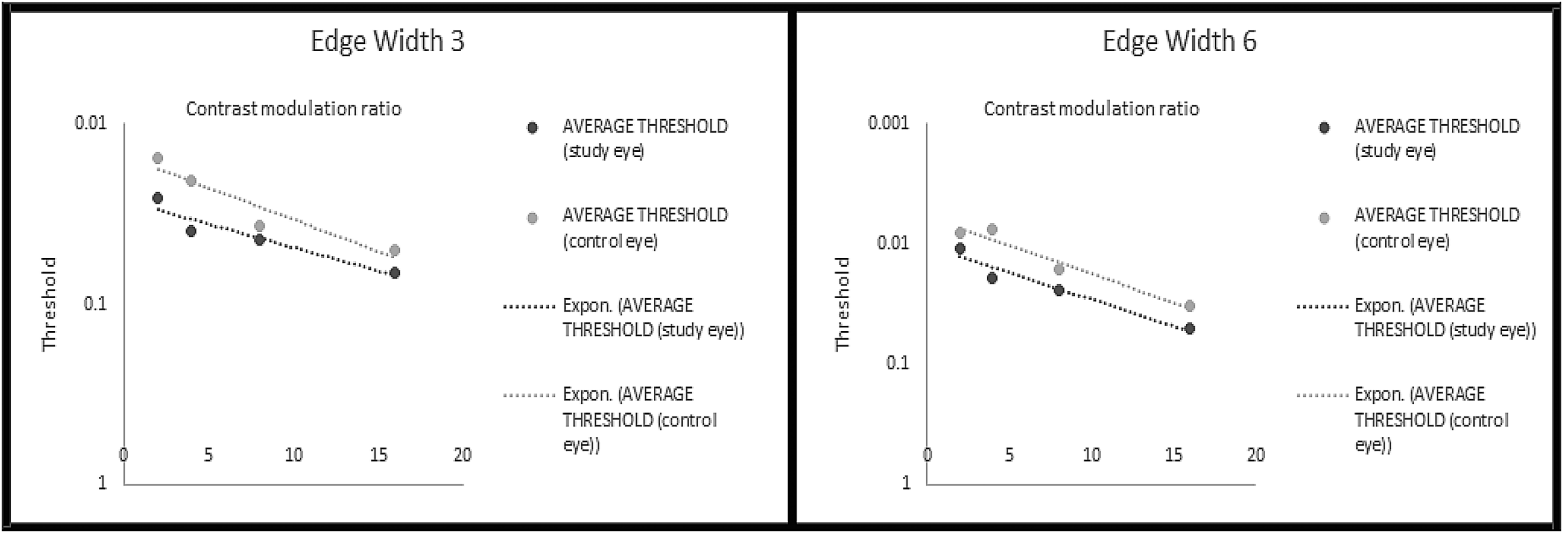
Comparison of average contrast sensitivity thresholds for RVO versus control eyes for the MDS assessment. Comparison of average contrast sensitivity thresholds (y-axis) for RVO (*n* = 20) versus control eyes (*n* =20) as a function of contrast modulation ratio (x-axis) for the MDS assessment. Four contrast modulation ratios were utilized (2, 4, 8, and 16). Threshold values are plotted logarithmically and inversely on the y-axis. Best-fit lines: exponential fitted regressions (Excel's GROWTH function) for each set of four averaged data points from study and control eyes. *Left:* MDS results from diamond edge width angle of 3 minutes. *Right:* MDS results from diamond edge width angle of 6 minutes.

The average PR assessment data is presented in Table 3 for the 20 study eyes and the 20 control eyes. There was a statistically significant difference (*P* value = 0.0001) between the log CS scores of the treated RVO eyes as compared to the controls (mean log CS of RVO eyes = 1.53 and mean log CS score of control eyes = 1.695).

### Subanalyses of RVO Study Eyes

As shown in Table 4, treated RVO eyes were delineated based on their classification of CRVO (*n* = 9) versus BRVO (*n* = 11) and their CFT, BCVA, α, β, and PR log CS scores were compared. No significant difference was found in CFT, BCVA, overall threshold levels, threshold slopes, and PR log CS scores when comparing CRVO study eyes to BRVO study eyes at edge-widths three and six.

**Table 4.** Subanalysis Comparing CRVO Study Eyes to BRVO Study Eyes

	CRVO	BRVO	*P* Value
**CFT**			
Mean (SD)	288.89 (30.25)	262 (37.83)	0.1015
**LogMAR BCVA**			
Mean (SD)	0.078 (0.097)	0.086 (0.075)	0.8439
**Pelli-Robson**			
Mean (SD)	1.583 (0.214)	1.473 (0.113)	0.1539
**MDS Edge-width 3 (mean)**			
α	0.035	0.021	0.5696
β	0.096	0.126	0.3921
**Edge-width 6 (mean)**			
α	0.010	0.010	0.9555
β	0.010	0.109	0.3664

The symbol α represents the threshold level for the threshold vs. contrast ratio curve and β represents the slope of the threshold versus contrast ratio curve.

^*^Indicates that the *P* value is significant and < 0.05.

CFT, central foveal thickness.

Furthermore, a subanalysis was performed comparing treated RVO eyes with either subretinal or intraretinal fluid (*n* = 9) to study RVO eyes with no fluid (*n* = 11; Table 5). The parameters being compared were the same as in the CRVO versus BRVO subanalysis. No significant difference was found in CFT, BCVA, overall threshold levels, threshold slopes, and PR log CS scores when comparing RVO eyes with fluid to those without.

**Table 5.** Subanalysis Comparing Study Eyes With Subretinal or Intraretinal Fluid to Study Eyes With no Fluid

	Fluid	No Fluid	*P* Value
**CFT**			
Mean (SD)	268.78 (49.47)	278.46 (22.74)	0.5687
**LogMAR BCVA**			
Mean (SD)	0.053 (0.068)	0.105 (0.091)	0.1732
**Pelli-Robson**			
Mean (SD)	1.567 (0.170)	1.500 (0.177)	0.4052
**MDS Edge-width 3 (mean)**			
α	0.022	0.032	0.6694
β	0.089	0.132	0.2128
**Edge-width 6 (mean)**			
α	0.009	0.011	0.7684
β	0.109	0.134	0.4654

The symbol α represents the threshold level for the threshold vs. contrast ratio curve and β represents the slope of the threshold vs. contrast ratio curve.

^*^Indicates that the *P* value is significant and < 0.05.

CFT, central foveal thickness.

### Comparison of RVO Non-Study Eyes to Normative Data

In Table 6, control eyes (*n* = 20) from the RVO patient population were compared to normative data on healthy eyes (*n* = 20) from patients with no ocular diseases. The VA (logMAR BCVA) and CS (measured via PR and MDS) of these two groups of eyes were compared. The PR demonstrated a significant difference between the RVO control eyes and healthy eyes with no ocular diseases (*P* value = 0.01). No significant differences were observed between the RVO control eyes and healthy eyes with respect to logMAR BCVA, α, and β values.

**Table 6.** Subanalysis Comparing Eyes From Patients With no Ocular Diseases to RVO Control Eyes

	No Ocular Disease	RVO Control	*P* Value
**LogMAR BCVA**			
Mean (SD)	0.048 (0.053)	0.082 (0.084)	0.131
**Pelli-Robson**			
Mean (SD)	1.575 (0.142)	1.695 (0.139)	0.01^*^
**MDS Edge-width 3**			
α	0.023 (0.063)	0.014 (0.023)	0.285
β	0.109 (0.076)	0.111 (0.055)	0.461
**Edge-width 6**			
α	0.005 (0.007)	0.006 (0.010)	0.534
β	0.128 (0.066)	0.127 (0.068)	0.954

The symbol α represents the threshold level for the threshold vs. contrast ratio curve and β represents the slope of the threshold vs. contrast ratio curve.

* Indicates that the *P* value is significant and < 0.05.

## Discussion

In this study, we examined CS in patients with RVO who were receiving anti-VEGF treatment utilizing both the PR and a novel MDS assessment. With regard to PR results, our study showed treated RVO eyes performed significantly worse on the PR as compared to the control eyes. Our result corroborates previously published data highlighting the impact of retinal diseases on vision.^6^^,^^7^^,^^24^ Notably, although these patients had decreased CS in their RVO eyes, the patients had excellent overall visual quality of life as determined by the VFQ-25.

The MDS assessment demonstrated no differences between study and control eyes at an edge-width of 3 MOA; however, differences were observed (in overall threshold but not adaptability of the visual contrast system) using a larger edge-width parameter of 6 MOA. This finding could be explained by baseline differences between the control and study eyes. As described in Table 2, the control eyes in this study had significantly higher BCVA as compared to the treated RVO eyes. It is plausible that taking the MDS assessment at a smaller edge-width of 3 MOA made visual acuity a confounding variable whereas at 6 MOA, VA deficiencies were no longer impacting performance on the MDS assessment. At an edge-width of 6 MOA, performance on the MDS assessment was more dependent on CS as compared to edge-width 3, which may explain why the results were more significant.

The data from the MDS assessment at edge-width six corroborates contrast threshold results from the PR assessment. Both assessments identify impairments in overall CS threshold for RVO eyes as compared to controls, which indicates that the α parameter from the MDS assessment may serve as a reliable parameter to quantify CS thresholds. Of note, the findings from PR were more significant, which could be explained by increased variability of the MDS data due to its overall contrast threshold level (α) parameter being dependent on a log-linear regression of four separate contrast threshold values. Furthermore, because the MDS utilizes a dynamic CS test region as compared to PR's static field, differences in the robustness of the data are expected.

Because RVO is a disease that results from systemic vascular dysfunction, the visual performance of control eyes (no vein occlusions) in patients with RVO was compared to healthy patients from another study conducted by Squires et al. that also utilized the MDS assessment (Squires N, et al. 2020 ARVO E-abstract 3366503). In that analysis, conflicting results were obtained. VA was not significantly different between the two groups. The MDS assessment showed no difference in CS between the RVO control eyes and healthy eyes; however, the PR did demonstrate a significant difference between these two groups. The MDS and Snellen results suggest that RVO control eyes are equivalent to healthy eyes in normal patients; however, the PR introduces a possibility that even control eyes in patients with RVO may have altered CS compared to healthy eyes in normal patients. Further study is indicated here to further characterize the potential impact of systemic vascular dysfunction on CS.

Subanalyses were also performed to examine the effect of subretinal or intraretinal fluid. It is logical to expect that retinal fluid, as detected by OCT, may have a detrimental impact on vision. However, our data suggest that the presence of retinal fluid in patients with treated RVO eyes had no significant correlation with changes in VA or CS (as measured by PR and MDS) compared to eyes without fluid.

With regard to quantifying adaptability of the visual contrast system (β) using the MDS, there is no existing gold standard to compare results. Taken at face value, results from the β parameter from the MDS at either edge-width suggest that treated patients with RVO do not have impairments in the adaptability of their visual system as compared for controls. This suggests that the mechanism by which RVO and its ocular sequelae lead to CS impairments does not involve reducing the visual system's ability to sensitize or desensitize itself to light levels or contrast gain.

The mechanism underlying visual system contrast encoding has been postulated to occur through two processing streams, the magnocellular (MC) and parvocellular (PC) pathways.^25^^,^^26^ The MC pathway has been demonstrated to have a high contrast gain and is able to approach saturation at relatively low levels of contrast. The MC pathway likely plays a significant role in modulating the adaptability of the contrast system because it is critical for detecting and discriminating briefly presented patterns with low contrast. The PC pathway, on the other hand, has a gradual linear contrast response that functions at higher contrast levels and is thought to mediate visual resolution, suggesting that the PC pathway could play a primary role in establishing an eye's contrast threshold level.^27^ Therefore, we suggest that the PR assessment may be particularly suited to characterizing the PC pathway of the visual contrast system whereas the MDS assessment can characterize both the PC and MC pathways. Based on the results from the MDS assessment, we postulate that RVO spares the MC pathway (evidenced by insignificant differences in β between study eyes and controls) but damages the PC pathway (evidenced by differences in overall contrast threshold between study eyes and controls per MDS and PR).

It is possible that the standard of care for RVO through anti-VEGF therapy is masking or reversing any adaptability impairments caused by RVO. The lack of CS adaptability impairments in patients with RVO may be partly responsible for their high quality of life, as evidenced by the VFQ-25 data.

The PR and the MDS are both assessments of CS; however, the MDS has many potential advantages. The PR is a well-established technique with demonstrated utility, but the technique has been criticized because it requires patients to have the ability to recognize letters.^28^ Furthermore, the PR can be influenced by lighting, reflections, and fading of the charts, and with fixed letters on a chart, a learning effect with repeated testing has been observed.^29^^,^^30^ Last, CS cannot be adequately quantified by PR's log CS threshold because differences between healthy and unhealthy visual systems that do not reveal themselves off of a steady state background (i.e. the white background of the PR) may do so with noise, pedestals, or other super-threshold additions that challenges the visual system.^31^^,^^32^

The MDS accounts for many of the aforementioned disadvantages of the PR. The MDS utilizes varying edge-widths to assess CS at varying levels of VA and does not require that the patients have the ability to identify letters. The MDS is displayed on a monitor, thus obviating concerns about lighting, reflections, and fading, although perhaps introducing new concerns about monitor uniformity and calibration. Additionally, the MDS challenges individuals to extract contrast from multiple luminance modulations (contrast ratio) above their threshold levels, thereby measuring a larger range of super threshold responses than the PR and to quantify adaptability. With these advantages, the MDS is a flexible digital tool that can be easily modified, distributed, and may even provide utility in domains beyond the reach of the PR, such as telemedicine.

A disadvantage to the MDS is that it is too lengthy for routine use, especially in comparison to the PR. However, the MDS assessment can be shortened. Because this is a pilot study for the MDS, a large response range was assessed. However, the MDS can conceivably be optimized to a single screen element, suitable for clinical environments.

A limitation of this study's design is that the study population consisted of individuals with excellent visual quality of life and was receiving long-term anti-VEGF therapy. As such, this study population may not be representative of the general population of patients being treated for RVO; therefore, further CS testing should be conducted on patients who are more recently diagnosed with RVO or before they began any anti-VEGF treatment. A further limitation is the sample size of the study. With 40 eyes included for analysis, this study's data lacked the power required for robust subanalyses. One of the research subjects recruited for this study had bilateral 3+ cataracts, although our protocol set an inclusion criteria limit of 2+ or lower cataracts. However, the enrolling investigator had identified no asymmetry in visual significance, in this case.

Currently, there is no gold standard for evaluating the adaptability of the visual contrast system; therefore, the β parameter of the MDS assessment does not have another clinical assessment-based parameter to which to be compared. Further data should be gathered on patients, both healthy and diseased, to establish the reliability of the β output parameter. Additionally, other retinal disease states, such as age-related macular degeneration, diabetic retinopathy, and central serous retinopathy, should be evaluated using both the MDS assessment to quantify changes in contrast threshold or contrast adaptability. Notably, previous research on contrast adaptation utilized electrophysiology studies, such as pattern electroretinograms and visually evoked potentials to help characterize changes in contrast gain adaptation.^33^^–^^35^ To better understand the mechanism of contrast adaptability changes in disease states, electrophysiology studies (pattern electroretinograms and visually evoked potentials) should be conducted in combination with the MDS contrast sensitivity assessment.

To address some of these limitations, follow-up studies are being conducted at Northwestern's Department of Ophthalmology. Both MDS and PR data are being collected on healthy patients and patients with other retinal diseases, such as age-related macular degeneration.
